# Wall Shear Stress Directional Abnormalities in BAV Aortas: Toward a New Hemodynamic Predictor of Aortopathy?

**DOI:** 10.3389/fphys.2018.00993

**Published:** 2018-08-14

**Authors:** Janet Liu, Jason A. Shar, Philippe Sucosky

**Affiliations:** Department of Mechanical and Materials Engineering, Wright State University, Dayton, OH, United States

**Keywords:** bicuspid aortic valve, wall shear stress, aortopathy, aorta, hemodynamics, helicity, directionality

## Abstract

The bicuspid aortic valve (BAV) generates wall shear stress (WSS) abnormalities in the ascending aorta (AA) that may be responsible for the high prevalence of aortopathy in BAV patients. While previous studies have analyzed the magnitude and oscillatory characteristics of the total or streamwise WSS in BAV AAs, the assessment of the circumferential component is lacking despite its expected significance in this highly helical flow environment. This gap may have hampered the identification of a robust hemodynamic predictor of BAV aortopathy. The objective of this study was to perform a global and component-specific assessment of WSS magnitude, oscillatory and directional characteristics in BAV AAs. The WSS environments were computed in the proximal and middle convexity of tricuspid aortic valve (TAV) and BAV AAs using our previous valve-aorta fluid-structure interaction (FSI) models. Component-specific WSS characteristics were investigated in terms of temporal shear magnitude (TSM) and oscillatory shear index (OSI). WSS directionality was quantified in terms of mean WSS vector magnitude and angle, and angular dispersion index (D_α_). Local WSS magnitude and multidirectionality were captured in a new shear magnitude and directionality index (SMDI) calculated as the product of the mean WSS magnitude and D_α_. BAVs subjected the AA to circumferential TSM overloads (2.4-fold increase vs. TAV). TAV and BAV AAs exhibited a unidirectional circumferential WSS (OSI < 0.04) and an increasingly unidirectional longitudinal WSS between the proximal (OSI > 0.21) and middle (OSI < 0.07) sections. BAVs generated mean WSS vectors skewed toward the anterior wall and WSS angular distributions exhibiting decreased uniformity in the proximal AA (0.27-point increase in D_α_ vs. TAV). SMDI was elevated in all BAV AAs but peaked in the proximal LR-BAV AA (3.6-fold increase vs. TAV) and in the middle RN-BAV AA (1.6-fold increase vs. TAV). This analysis demonstrates the significance of the circumferential WSS component and the existence of substantial WSS directional abnormalities in BAV AAs. SMDI abnormality distributions in BAV AAs follow the morphotype-dependent occurrence of dilation in BAV AAs, suggesting the predictive potential of this metric for BAV aortopathy.

## Introduction

The bicuspid aortic valve (BAV) is a congenital heart valve defect consisting of the formation of two functional leaflets instead of three. BAVs exist in different phenotypes: left- and right-coronary leaflet fusion (LR-BAV), right- and non-coronary leaflet fusion (RN-BAV), and non- and left-coronary leaflet fusion (NL-BAV) (Sievers and Schmidtke, 2007). BAV patients are exposed to increased risk of secondary aortopathies such as aortic dilation, aneurysm, and dissection, which typically develop in the convexity of the ascending aorta (AA) (Braverman et al., 2005; Tzemos et al., 2008; Losenno et al., 2012). Experimental (Seaman et al., 2014; Saikrishnan et al., 2015; McNally et al., 2017), computational (Cao and Sucosky, 2015; Cao et al., 2017; Kimura et al., 2017), and clinical (Barker et al., 2010; Bissell et al., 2013; Mahadevia et al., 2014) studies have demonstrated that BAVs can cause flow abnormalities in the AA, marked by increased skewness, helicity, and wall shear stress (WSS) overloads on the convexity of the aortic wall. The apparent colocalization of WSS abnormalities and dilation patterns on the convexity of BAV aortas (Cotrufo et al., 2005; Della Corte et al., 2008) has suggested a role for hemodynamics in the etiology of BAV aortopathy (Atkins and Sucosky, 2014; Michelena et al., 2014; Mathieu et al., 2015; Yassine et al., 2017) and has motivated further investigations of the local WSS environment.

Previous hemodynamic analyses performed in BAV AAs have focused on quantifying bulk flow shear magnitude (McNally et al., 2017), temporal shear magnitude (TSM) and oscillatory shear index (OSI) in the streamwise (Barker et al., 2010, 2012; Cao and Sucosky, 2015; Cao et al., 2017) and circumferential directions (Meierhofer et al., 2013; Piatti et al., 2017). While these WSS metrics have provided new insights into the morphotype-dependent regional stress abnormalities in BAV aortas, they lack any predictive capability for BAV aortopathy. This could be due to the inability of those metrics to fully capture the impact of increased flow helicity on the WSS environment, which could be instrumental to the pathogenesis of BAV aortopathy.

The elucidation of the potential involvement of BAV flow helicity in aortopathy requires a more detailed and spatially resolved assessment of the WSS directional characteristics in BAV AAs. In pursuit of this goal, the objective of the present study was to compare the component-specific magnitude and directional characteristics of the WSS vector in TAV and BAV AAs. The results of this analysis were used to investigate a novel hemodynamic metric capturing the effects of WSS magnitude and directionality abnormalities, with potential predictive capabilities for BAV aortopathy.

## Materials and methods

### Computational models

**Figure 1:**
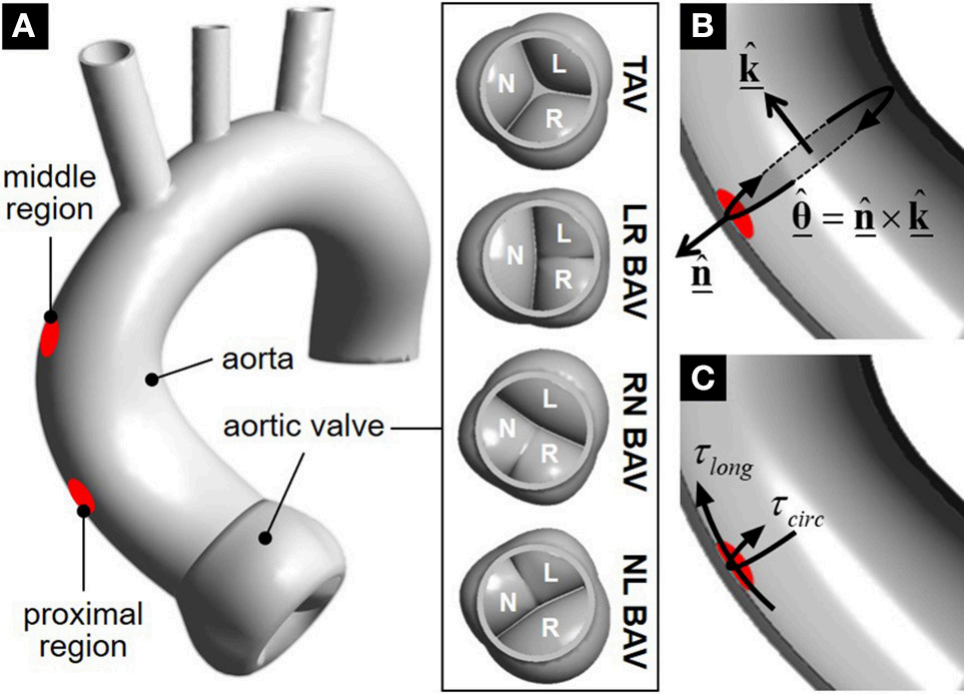
Geometrical models and WSS extraction: **(A)** valve-aorta models and WSS interrogation regions; **(B)** longitudinal ($\hat{\underset{-}{k}}$) and circumferential ($\hat{\underset{-}{\theta }}$) direction definitions; and **(C)** extracted WSS components.

Instantaneous WSS characteristics were extracted from our previous fluid-structure interaction (FSI) valve-aorta models (Cao et al., 2017). Briefly, the models consisted of four aortic valve geometries (TAV, LR-BAV, NL-BAV, RN-BAV) connected to an idealized aorta reconstructed from a series of computed tomography images of a human aorta (Figure 1A). Valve leaflet and aortic wall tissues were modeled using a three-parameter Mooney-Rivlin model and a linear elastic model, respectively, calibrated with respect to published tensile test data on valvular and aortic tissue (Cao et al., 2016, 2017). Two-way FSI simulations were performed in ANSYS 18.0 Fluent, Mechanical ADPL and System Coupling (ANSYS Inc.) using the arbitrary Lagrangian-Eulerian (ALE) approach. The governing equations consisted of the momentum and continuity equations for the fluid domain, the momentum equation for the structural domain, and three coupling conditions enforcing continuity of displacements, velocities and tractions at the fluid-structure interface. All models were subjected to the same physiologic transvalvular pressure waveform (0–12 mmHg, 2:1 diastolic/systolic ratio). The capability of this FSI approach in reproducing the native features of aortic root and AA dynamics has been evidenced previously (Sturla et al., 2013) and the model has been recently validated against *in vitro* particle-image velocimetry measurements performed in similar AA geometries (McNally et al., 2017).

### WSS components extraction

The instantaneous WSS vector $\underset{-}{\tau }$ as well as its longitudinal and circumferential components (τ_*long*_ and τ_*circ*_, respectively) were captured over two circular regions (7 mm in diameter) located 28 and 47 mm above the sinotubular junction in the dilation-prone convexity of the proximal and middle aortic wall, respectively (Figure 1A). The longitudinal direction was defined as the normal unit vector $\hat{\underset{-}{k}}$ to the cross section of the aorta intersecting each target region on the wall (Figure 1B). The circumferential unit vector $\hat{\underset{-}{\theta }}$ was obtained as the cross product between the outward normal unit vector $\hat{\underset{-}{\text{n}}}$ to the target region and the longitudinal unit vector:

$$\begin{array}{l} \hat{\underset{–}{\theta }}=\hat{\underset{–}{n}}\times \hat{\underset{–}{k}} \end{array}$$

The local longitudinal and circumferential WSS components were obtained by calculating the dot product between the local WSS vector and the respective unit vector in each direction (Figure 1C):

$$\begin{array}{l} \tau_{\text{long}}=\underline{\tau }\cdot \underline{\hat{k}} \end{array}$$

and

$$\begin{array}{l} \tau_{circ}=\underline{\tau }\cdot \underline{\hat{\theta }} \end{array}$$

### Component-specific WSS magnitude and oscillatory characteristics

To investigate the respective contribution of each WSS component, time-averaged longitudinal and circumferential WSS magnitudes were quantified in terms of temporal shear magnitude (TSM),

$$\begin{array}{l} TSM_{i}=\frac{1}{T}\underset{0}{\overset{T}{\int }}|\tau_{i}|\;dt \end{array}$$

where *i* = [*long, circ*] and *T* is the duration of one cardiac cycle. The oscillatory nature of both WSS components along their respective axis was further quantified in terms of the oscillatory shear index (OSI),

$$\begin{array}{l} OSI_{i}=\frac{1}{2}\left(1-\frac{\left|\int_{0}^{T}\tau_{i}\;dt\right|}{\int_{0}^{T}|\tau_{i}|\;dt}\right) \end{array}$$

where a value of 0 indicates a purely unidirectional WSS and a value of 0.5 indicates a purely oscillatory bidirectional WSS.

### WSS directional characteristics

At each instant of time, the angle between the instantaneous WSS vector and the longitudinal direction was quantified as:

$$\begin{array}{l} \alpha =\{\begin{array}{c} \cos^{-1}\;\left(\frac{\underline{\tau }\cdot \underline{\hat{k}}}{\left|\underline{\tau }\right|}\right)\text{ if }\underline{\tau }\cdot \hat{\underline{\theta }}>0\\ 360^{{}^{\circ}}-\cos^{-1}\left(\frac{\underline{\tau }\cdot \hat{\underline{k}}}{\left|\underline{\tau }\right|}\right)\text{ if }\underline{\tau }\cdot \hat{\underline{\theta }}<0 \end{array} \end{array}$$

This piecewise formulation was implemented to obtain the correct value of the WSS angle over the full trigonometric range (i.e., from 0 to 360°). Temporal changes in WSS magnitude and directionality were then investigated by tracing the tip of the instantaneous WSS vector $\underset{-}{\tau }$ in a polar plot over one cardiac cycle.

WSS multidirectionality characteristics were also assessed on time-average basis using directional statistics theory (Fisher, 1995). The mean WSS vector over each interrogation region was determined by calculating the mean WSS magnitude $\bar{\tau }$ and the mean WSS angle $\bar{\alpha }$. The mean WSS magnitude over one cycle (*T*) was calculated as

$$\begin{array}{l} \bar{\tau }=\frac{1}{T}\underset{0}{\overset{T}{\int }}|\underset{-}{\tau }|\;dt \end{array}$$

The mean angular orientation $\bar{\alpha }$ of the WSS vector relative to the longitudinal direction over one cardiac cycle was determined by first calculating its absolute value,

$$\begin{array}{l} \tilde{\alpha }=|\tan^{-1}\left(\frac{\bar{\sin}\;\alpha }{\bar{\cos}\;\alpha }\right)| \end{array}$$

where the time-averaged cosine and sine of the WSS vector angle were calculated as

$$\begin{array}{l} \{\begin{array}{c} \bar{\sin}\;\alpha =\frac{1}{T}\underset{0}{\overset{T}{\int }}\sin\;\alpha dt\\ \bar{\cos}\;\alpha =\frac{1}{T}\underset{0}{\overset{T}{\int }}\cos\;\alpha dt \end{array} \end{array}$$

and then determining its correct quadrant by implementing the following piecewise formulation,

$$\{\begin{array}{lll} \bar{\alpha }=\tilde{\alpha } & \text{if} & \bar{\sin}\;\alpha >0\text{ and }\bar{\cos}\;\alpha >0\\ \bar{\alpha }=180{}^{\circ}-\tilde{\alpha } & \text{if} & \bar{\sin}\;\alpha >0\text{ and }\bar{\cos}\;\alpha <0\\ \bar{\alpha }=180{}^{\circ}+\tilde{\alpha } & \text{if} & \bar{\sin}\;\alpha <0\text{ and }\bar{\cos}\;\alpha <0\\ \bar{\alpha }=360{}^{\circ}-\tilde{\alpha } & \text{if} & \bar{\sin}\;\alpha <0\text{ and }\bar{\cos}\;\alpha >0 \end{array}$$

The uniformity of the angular distribution of the WSS vector was also investigated in terms of the angular dispersion index,

$$\begin{array}{l} D_{\alpha }={\left[{\bar{\cos}}^{2}\alpha +{\bar{\sin}}^{2}\alpha \right]}^{1/2} \end{array}$$

which ranges from 0 (uniform, equi-angularly spaced WSS vector distribution) to 1 (concentration of WSS vectors in one unique direction).

### Shear magnitude and directionality index (SMDI)

In an effort to account for the combined effects of flow helicity on WSS magnitude and angular distribution, which are not fully captured by current WSS metrics, a new shear magnitude and directionality index (SMDI) was proposed and defined as

$$\begin{array}{l} SMDI=\bar{\tau }\times D_{\alpha } \end{array}$$

By quantifying the cumulative effects of WSS multidirectionality and overload, this metric is expected to provide a more representative assessment of the local degree of hemodynamic abnormality in BAV AAs. This metric will be computed in the proximal and middle sections of TAV and BAV AAs.

## Results

### Longitudinal and circumferential WSS characteristics

**Figure 2:**
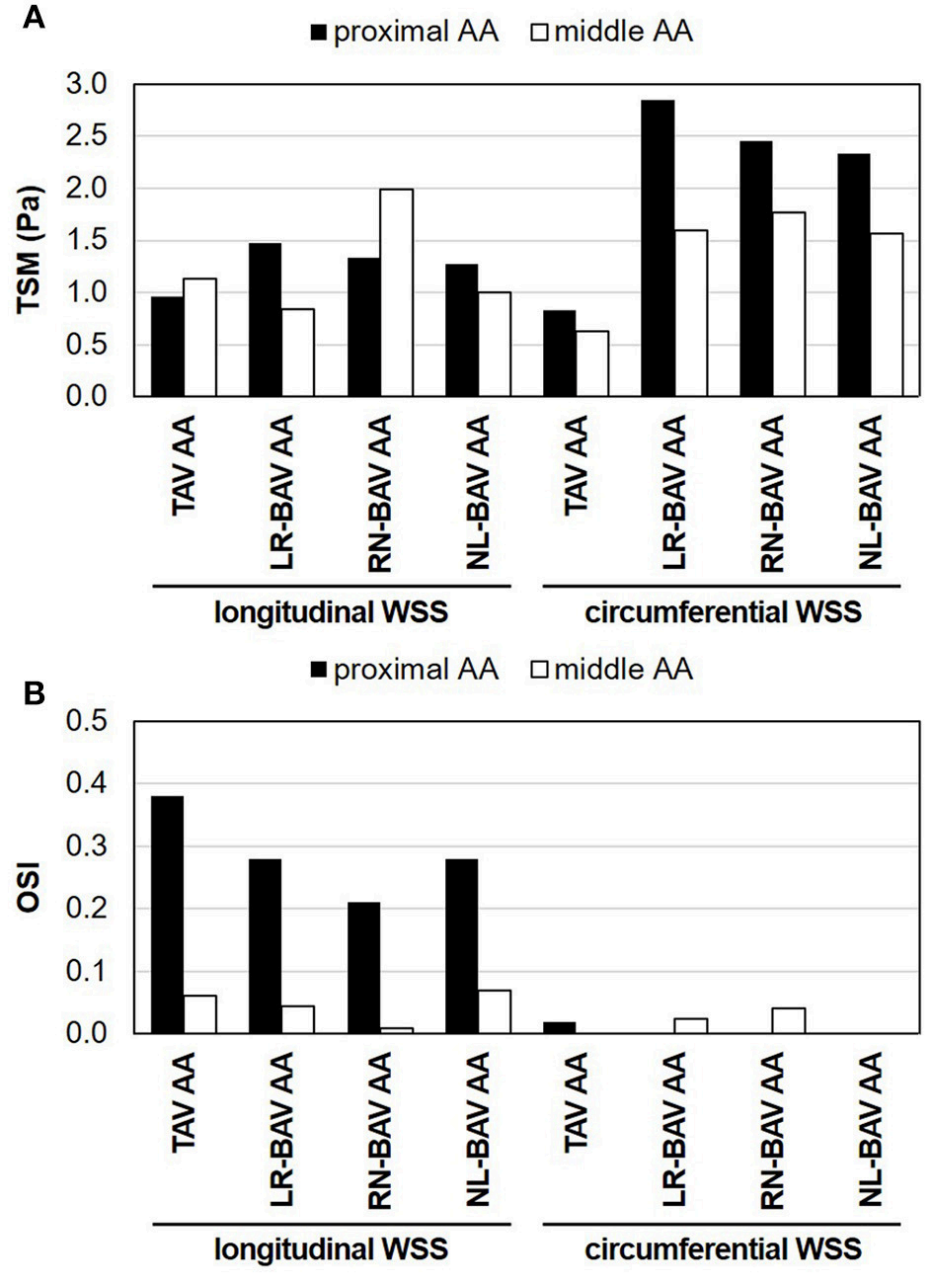
Component-specific WSS magnitude and oscillatory characteristics in the proximal and middle convexity of TAV and BAV AAs: **(A)** TSM; and **(B)** OSI.

The comparison of the component-specific TSM between all valves indicates the existence of large WSS overloads in BAV AAs (Figure 2A). The most substantial overload affects the circumferential WSS component in the proximal AA (1.8- to 2.4-fold increase vs. TAV AA). The longitudinal TSM exhibits more dependence on the BAV morphotype and less deviation from that predicted in the TAV AA (<1.8-fold increase vs. TAV). Lastly, while the longitudinal and circumferential TSM are essentially similar in the proximal TAV AA (13% difference between the two components), the longitudinal TSM becomes increasingly dominant in the middle AA (44% difference). In contrast, the increased BAV flow helicity tends to generate a WSS environment more sharply dominated by the circumferential WSS component (1.6- to 1.9-fold larger than longitudinal TSM).

The analysis of the OSI reveals contrasted WSS oscillatory characteristics along the longitudinal and circumferential directions (Figure 2B). Regardless of the valve anatomy, the longitudinal WSS component is strongly bidirectional in the proximal AA (OSI > 0.21), but nearly purely unidirectional in the middle AA (OSI < 0.07). This contrasts with the circumferential WSS component, which remains essentially unidirectional (OSI < 0.04) and is weakly impacted by the AA location or valve anatomy. While those global characteristics are common to TAV and BAV AAs, BAVs tend to attenuate the degree of longitudinal WSS oscillation in the proximal AA relative to the TAV (0.10–0.17-point reduction in longitudinal OSI vs. TAV).

### Instantaneous and mean WSS directional characteristics

**Figure 3:**
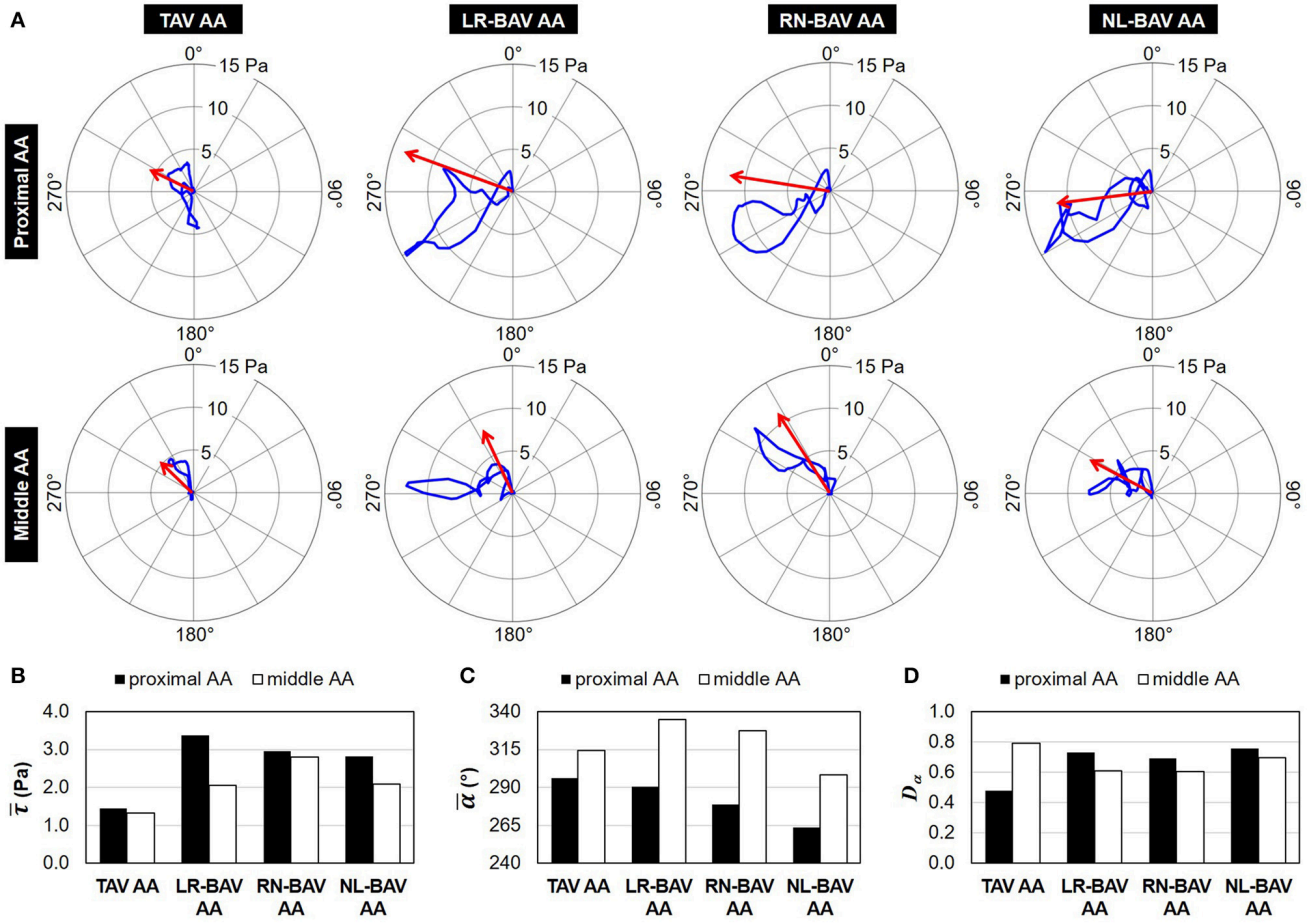
WSS directional characteristics: **(A)** polar plots showing the trace of the tip of the WSS vector (blue) and the mean WSS vector (red) over one cardiac cycle (0° angle: longitudinal direction; mean WSS vector scale: 4×); **(B)** mean WSS magnitude; **(C)** mean WSS angle relative to the longitudinal direction; and **(D)** angular dispersion index computed in the proximal and middle convexity of TAV and BAV AAs.

Animations of the WSS vectors predicted in all models are provided as online Supplementary Material (Supplementary Video 1). The temporal traces of the tip of the instantaneous WSS vector and the mean WSS vectors (magnitude: $\bar{\tau }$; angle: $\bar{\alpha }$) are reported in Figure 3A. Both TAV and BAVs generate traces that remain in the left half of the polar plot (i.e., anterior wall region). While the traces captured in the proximal AA cross the circumferential axis (90°-270° line), those captured in the middle AA remain in the upper half of the plot.

The comparison of the mean WSS vector magnitude demonstrates the existence of substantial WSS overloads in all BAV AAs (1.5- to 2.3-fold increase vs. TAV AA) that peak in the proximal wall region (Figure 3B). On the other hand, the mean WSS magnitude is weakly impacted by the BAV morphotype (<12% difference between BAVs in the proximal AA, <26% difference in the middle AA). With the exception of the NL-BAV, which generates a mean WSS vector pointing slightly backward ($\bar{\alpha }=$ 263°) in the proximal AA, the orientation of the mean WSS vector is forward and toward the anterior wall ($\bar{\alpha }>$ 270°) in all other cases (Figure 3C). The presence of a BAV tends to increase the circumferential alignment of the mean WSS vector in the proximal AA ($263^{{}^{\circ}}<\bar{\alpha }<$ 290°), suggesting the greater impact of the circumferential WSS component in BAV anatomies relative to the TAV in that region. In all models, the progressive development of the flow between the proximal and middle sections is accompanied by a net decrease in circumferential flow motion as indicated by the increased alignment of the mean WSS vector toward the longitudinal direction (proximal AA: $263^{{}^{\circ}}<\bar{\alpha }<$ 296°; middle AA: $298^{{}^{\circ}}<\bar{\alpha }<$ 335°).

The comparison of the uniformity of the WSS angular distribution in TAV and BAV AAs reveals opposite trends in the proximal and middle sections (Figure 3D). The TAV AA WSS environment is characterized by a decreasing degree of WSS angular uniformity between the proximal and middle AA (0.31-point increase in *D*_α_ between the two sections). In contrast, BAVs tend to generate a slightly more uniform WSS angular distribution in the middle AA than in the proximal AA (0.06 to 0.12-point decrease in *D*_α_ between the two sections). In addition, while BAVs generate less WSS angular uniformity than the TAV in the proximal AA (0.21–0.27-point increase in *D*_α_ vs. TAV), the trend is reversed in the middle AA (0.10–0.19-point decrease in *D*_α_ vs. TAV).

### SMDI

**Figure 4:**
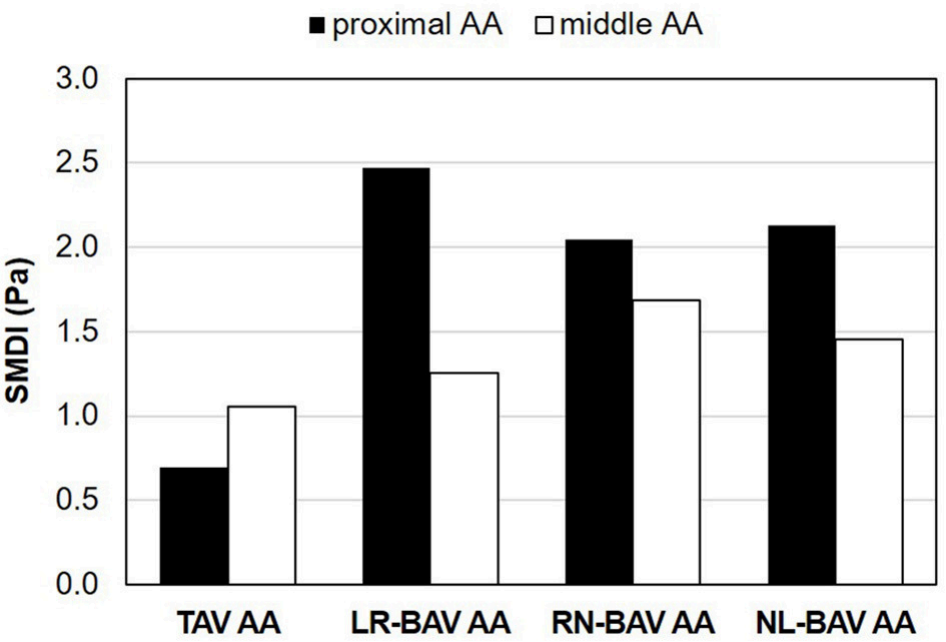
SMDI predictions in the proximal and middle convexity of TAV and BAV AAs.

The comparison of the SMDI in TAV and BAV AAs is reported in Figure 4. Baseline levels computed in the proximal and middle TAV AA reveal a normal SMDI range between 0.69 and 1.05. In comparison, BAVs generated substantially larger SMDI values ranging between 1.25 and 2.47. The degree of SMDI abnormality computed in each AA section exhibited strong dependence on the BAV morphotype. In the proximal AA, the maximum degree of SMDI abnormality is generated by the LR-BAV (3.6-fold increase vs. TAV), followed by the NL- and RN-BAV (3.1 - and 3.0-fold increase, respectively, vs. TAV). In the middle AA, the RN-BAV generates the highest degree of abnormality (1.6-fold increase vs. TAV), followed by the NL- and LR-BAV (1.4- and 1.2-fold increase, respectively, vs. TAV).

## Discussion

In this study, the WSS environments generated by the TAV and different BAV morphotypes were analyzed in terms of global and component-specific magnitude and directional characteristics. A new SMDI metric integrating the magnitude and angular distribution uniformity of the local WSS vector was proposed and computed. This analysis reveals: (1) the domination of the BAV AA WSS by its circumferential component; (2) the existence of WSS overloads in BAV AAs associated with increased levels in circumferential WSS; (3) the existence of region-specific differences in angular WSS uniformity between TAV and BAV AAs; and (4) similarities between regional morphotype-dependent dilation patterns and the local degree in SMDI abnormality.

### Increased flow helicity is responsible for WSS overloads in BAV AAs

Previous *in vivo, in vitro* and computational studies have demonstrated the existence of WSS overloads in BAV AA regions prone to dilation (Barker et al., 2010, 2012; Hope et al., 2011; Bissell et al., 2013; Mahadevia et al., 2014; Seaman et al., 2014; Cao and Sucosky, 2015; Cao et al., 2017; McNally et al., 2017; Piatti et al., 2017). The present analysis on both the global and component-specific WSS magnitudes (as quantified by the mean WSS magnitude and component-specific TSM) suggests the stronger impact of the BAV anatomy on the circumferential WSS component than on the longitudinal component. This important result suggests that WSS overloads in BAV AAs stem from a transfer of flow momentum from the streamwise to the circumferential direction, which is consistent to the increased BAV flow helicity reported by many investigations. This observation confirms previous *in vivo* findings obtained by cardiac magnetic resonance (CMR) in the middle LR-BAV and RN-BAV AAs (Meierhofer et al., 2013), while extending them to the proximal AA section and the NL-BAV morphotype.

### BAVs attenuate WSS multidirectionality in the AA convexity

The present study introduced two novel strategies adopted from circular statistics theory to characterize WSS angular variations. First, the traces of the instantaneous WSS vector captured over one cardiac cycle in the AA convexity revealed the preferential orientation of the BAV WSS vector toward the anterior wall, which contrasted with the much broader angular range spanned by the TAV WSS vector. Second, the angular dispersion index was investigated to assess the uniformity of the WSS angular distribution and confirmed the susceptibility of the BAV WSS vector to align along a particular direction.

WSS directionality in BAV AAs has been analyzed historically in terms of general WSS vector orientation through the comparative magnitudes of the WSS components (Meierhofer et al., 2013; Piatti et al., 2017), or WSS bidirectionality along a given direction through OSI calculations (Cao and Sucosky, 2015; Cao et al., 2017; Piatti et al., 2017). Our OSI predictions, which indicated similar WSS oscillatory characteristics in TAV and BAV AAs, suggests the inability of this metric to capture the differences in WSS multidirectionality clearly evidenced by the WSS vector traces. Similarly, while the analysis of the mean WSS vector angle confirmed the preferential alignment of the BAV AA WSS vector along the circumferential direction, it did not provide any assessment of the directional range spanned by the WSS vector. Those computational results suggest the relevance of the angular dispersion index for the effective characterization of WSS multidirectionality.

### Comparison with previous *in vivo* studies and benefits of the computational approach

To our knowledge, two published *in vivo* studies have investigated the component-specific characteristics of the WSS in BAV AAs. One consisted of a prospective 4D CMR investigation of the time-averaged magnitude of the WSS vector and its components in the middle and distal AA of 18 LR- and RN-BAV patients without any other concomitant cardiovascular disease (Meierhofer et al., 2013). The study reported elevated total and circumferential WSS in the BAV population relative to the TAV group and suggested circumferential WSS as an indicator for BAV aortic dilation. More recently, another 4D CMR study provided the first *in vivo* assessment of longitudinal and circumferential TSM and OSI in 5 non-dilated BAV AAs (Piatti et al., 2017). The study revealed the existence of patient-specific WSS abnormalities in BAV AAs, a wider range of transversal WSS and the existence of the absence of substantial circumferential WSS oscillation. Our computational results are in agreement with these previous findings but also present several important benefits. First, as compared to clinical studies, the use of a computational approach permitted to eliminate any patient variability in flow conditions (e.g., cardiac output, transvalvular pressure) or anatomy (e.g., number of aortic sinuses, aortic diameter). As a result, all WSS differences described in the present study are only due to the different valve morphotypes implemented in the models. In addition, by investigating the WSS environment locally in the aorta convexity, our study complements the previous characterizations conducted in different aorta cross-sections (Meierhofer et al., 2013) or over the full aortic wall perimeter (Piatti et al., 2017), and provides new insights into WSS alterations in the disease-prone region of the aorta.

### SMDI as a potential hemodynamic marker of BAV aortopathy

Flow helicity and WSS magnitude have shown some degree of correlation with the local expression of aortopathy in BAV aortas (Hope et al., 2011; Barker et al., 2012; Bissell et al., 2013; Meierhofer et al., 2013; Mahadevia et al., 2014; Cao et al., 2017). The present study confirms this trend by demonstrating maximum WSS overloads in the proximal section of the LR-BAV AA and in the middle section of the RN-BAV AA, where dilation is typically observed with these morphotypes (Bauer et al., 2006; Kang et al., 2013). However, two limitations have prevented the use of these metrics as robust predictors of BAV aortopathy. First, elevated WSS is not a feature exclusive to BAV flow. Other conditions such as valvular stenosis can also subject the aortic wall to high WSS magnitude (van Ooij et al., 2017) without a demonstrated link to aortopathy (Boudoulas et al., 2015). Second, helicity is a bulk flow characteristic calculated in an entire section of the aorta and therefore, may not be representative of the local hemodynamic state of the aortic wall. As such, abnormalities in helicity reported in different aortic sections of BAV AAs (Faggiano et al., 2013; Cao et al., 2017) do not permit to explain the asymmetry of dilation in BAV AAs. These limitations justify the need for a hemodynamic metric characterizing the local stress environment experienced by the aortic endothelium. The new SMDI proposed in this study aims at filling this gap by quantifying at the same time the local multidirectionality or the WSS and its magnitude. SMDI predictions not only exhibited elevated levels in all BAV AAs as compared to the TAV AA, they also correlated with the local expression of aortopathy (maximum SMDI in proximal AA generated by LR-BAV; maximum SMDI in middle AA generated by RN-BAV).

### Potential impact on aortic wall biology

Previous biological characterizations of aortic tissue excised from the wall convexity in BAV patients have revealed structural wall abnormalities including smooth muscle cell (SMC) apoptosis and depletion, elastic fiber degeneration and extracellular matrix remodeling (Bonderman et al., 1999; Fedak et al., 2003; Nataatmadja et al., 2003; Boyum et al., 2004; Ikonomidis et al., 2007; Tadros et al., 2009; Phillippi et al., 2014). Although these observations seem to align with the flow alterations evidenced in the same regions, the potential relationship between local hemodynamics and pathobiology has not been fully demonstrated yet. The response of aortic endothelial cells to BAV hemodynamics has been investigated *ex vivo* in shear stress devices enabling the replication of the native BAV AA WSS (Sucosky et al., 2008). These studies have suggested the susceptibility of BAV hemodynamic stresses to focally mediate aortic medial degradation in the convexity by upregulating matrix metalloproteinase expression and activity (Atkins et al., 2014, 2016). However, the current limitation of shear stress devices to WSS production along one single direction does not permit to elucidate the potential effect of WSS directionality, which has been shown to exhibit contrasted characteristics in TAV and BAV aortas in the present study. Ongoing work conducted in our laboratory on the design of a multidirectional WSS bioreactor is expected to address this limitation (Liu, 2018).

## Limitations

Several limitations must be discussed in the methodologies adopted for the study. First, the valve-aorta models used to perform the WSS analysis only provide an approximation of the native WSS environment. They implemented idealized geometries and boundary conditions that do not capture the range of patient variability, and material formulations that only approximate the anisotropic and non-linear mechanical response of the leaflets and the aortic wall to flow (Billiar and Sacks, 2000; Danpinid et al., 2010). In this context, the modeling of the aortic wall as a linear elastic material is a restrictive assumption. However, additional simulations performed to assess the impact of this assumption on the local hemodynamics revealed only a 0.2% difference in peak-systolic WSS in the proximal aortic convexity using a linear model and a more physiologic two-parameter Mooney Rivlin model (Ranga et al., 2007). This result confirms previous findings suggesting the appropriateness of the linear elastic material assumption to correctly capture the native aorta hemodynamics over the range of physiological intra-arterial pressures (Crosetto et al., 2011). In addition, while the new SMDI exhibited substantial differences between TAV and BAV AA, its sensitivity to the BAV morphotype in each AA section was more subtle. A possible explanation is the relatively small size of the regions where WSS was captured, which made the assessment of all flow metrics rather localized. While larger interrogation regions could have been used to discretize the entire AA into the same segments as those typically considered to define dilation patterns (i.e., root, tubular AA, distal AA and transverse arch Kang et al., 2013; Khoo et al., 2013), they would have made it more difficult to define a local longitudinal and circumferential directions given the drastic changes in wall curvature.

Lastly, although the new SMDI proposed in this study has the capability to quantify both WSS directionality and magnitude, and has shown some interesting similarities with the morphotype-dependent patterns of BAV dilation, this metric has not been validated experimentally as a predictor of BAV aortopathy. Further analyses are needed to investigate the robustness of this metric in actual BAV patient aortas. However, the benefits of the computational approach adopted in this study should not be underestimated. In fact, the investigation of hemodynamic predictors of BAV aortopathy is difficult to conduct *in vivo* due to the inherent patient-specificity of the flow that may challenge the identification of a robust universal metric. The unique ability of the computational strategy to isolate the impact of the valve geometry on the aortic stress state while eliminating any other patient-specific anatomic and hemodynamic variable constitutes the main strength of the modeling approach. Nevertheless, until the SMDI predictions reported in this computational study can be validated against *in vivo* measurements, the predictive power of this new hemodynamic index for BAV aortopathy should be considered with caution.

## Author contributions

JL and JAS analyzed the data and wrote the paper. PS conceived the work, analyzed the data and edited the paper.

### Conflict of interest statement

The authors declare that the research was conducted in the absence of any commercial or financial relationships that could be construed as a potential conflict of interest.
