# Supplementary material for: Wall Shear Stress Directional Abnormalities in BAV Aortas: Toward a New Hemodynamic Predictor of Aortopathy?
Source: Front Physiol. 2018 Aug 14;9:993. doi: 10.3389/fphys.2018.00993 (PMC6102585; doi:10.3389/fphys.2018.00993)
Supplement: Supplementary file 2 [file Data_Sheet_1.DOCX]

Supplementary Material

Wall Shear Stress Directional Abnormalities in BAV Aortas: Toward a New Hemodynamic Predictor of Aortopathy?

Janet Liu^1^, Jason A. Shar^1^, Philippe Sucosky^1*^

*** Correspondence:** Philippe Sucosky: philippe.sucosky@wright.edu

**Supplementary Video 1.** Temporal variations of the local WSS vector and surface-averaged WSS contour captured in the proximal and middle regions of TAV, LR-BAV, RN-BAV and NL-BAV AAs (playback speed: 0.25×).
